# Impact of relaxation training and sedative medication use on respiratory recovery in hospitalized patients with respiratory diseases: a retrospective observational study

**DOI:** 10.3389/fmed.2026.1743290

**Published:** 2026-04-17

**Authors:** Min Liang, Ziyu Song, Ke Gong

**Affiliations:** 1 Department of Psychosomatic Medicine, Hejiang People’s Hospital, Sichuan, China; 2 Department of Pharmacy, Hejiang People’s Hospital, Sichuan, China; 3 Department of Psychosomatic Medicine, The Affiliated Hospital, Southwest Medical University, Luzhou, Sichuan, China

**Keywords:** anxiety, relaxation training, respiratory recovery, retrospective study, sedative medication

## Abstract

**Background:**

Psychological distress and sedative medication exposure are common among hospitalized patients with respiratory diseases and may influence clinical recovery. However, evidence regarding the combined impact of relaxation training and medication use on respiratory outcomes remains limited. This study aimed to examine the association between relaxation training, sedative or psychotropic medication use, and respiratory recovery in patients hospitalized with respiratory diseases.

**Methods:**

A retrospective observational study was conducted using electronic medical records from Hejiang County People’s Hospital, Sichuan Province, China. Consecutive adults ( ≥ 18 years) admitted with a primary diagnosis of chronic obstructive pulmonary disease, pneumonia, asthma, bronchiectasis, or interstitial lung disease between January 2020 and January 2025 were included. Patients were categorized according to documented exposure to relaxation training during hospitalization. The primary outcome was respiratory recovery at discharge, defined a priori using objective criteria based on routinely documented records: successful discontinuation of invasive or non-invasive ventilatory support (if applicable), oxygen saturation ≥ 95% on room air for ≥ 4 h, respiratory rate 12–24 breaths/min at rest, and no evidence of ongoing respiratory failure (pH ≥ 7.35 and no worsening hypercapnia [PaCO_2_ not increasing compared with the most recent prior measurement] and/or PaO_2_ ≥ 60 mmHg on room air when an arterial blood gas test was available within 24 h before discharge). Variables associated with recovery were identified using univariate and multivariable logistic regression analyses. Model discrimination, calibration, and clinical utility were assessed by the concordance index (C-index), Hosmer–Lemeshow test, calibration plot, and decision curve analysis.

**Results:**

A total of 389 patients were included (mean age 63.3 ± 12.2 years; 58.1% male), of whom 158 (40.6%) received relaxation training. In multivariable analysis, younger age (OR = 0.96, 95% CI 0.94–0.98, *P* = 0.002), lower anxiety scores (OR = 0.87, 95% CI 0.81–0.93, *P* < 0.001), lower PaCO_2_ levels (OR = 0.94, 95% CI 0.91–0.97, *P* < 0.001), higher PaO_2_/FiO_2_ ratio (OR = 1.07 per 10-unit increase, 95% CI 1.03–1.12, *P* = 0.001), and lower CRP levels (OR = 0.98, 95% CI 0.97–0.99, *P* = 0.007) were independently associated with respiratory recovery. Participation in relaxation training significantly increased the odds of recovery (OR = 2.38, 95% CI 1.45–3.90, *P* < 0.001), whereas early benzodiazepine exposure within 72 h of admission reduced the likelihood of recovery (OR = 0.37, 95% CI 0.22–0.63, *P* < 0.001). The predictive model demonstrated good discrimination (C-index = 0.872, 95% CI 0.799–0.944) and satisfactory calibration (Hosmer–Lemeshow χ^2^ = 6.585, *P* = 0.582). Decision curve analysis indicated a higher net clinical benefit of the model across relevant probability thresholds.

**Conclusion:**

In this retrospective cohort of hospitalized patients with respiratory diseases, engagement in relaxation training was independently associated with improved respiratory recovery, whereas early sedative medication exposure predicted poorer outcomes. Psychological and physiological parameters jointly influenced recovery probability. These findings underscore the importance of integrating psychological relaxation interventions and cautious sedative management in the clinical care of respiratory patients.

## Introduction

Hospitalized patients with respiratory diseases—such as chronic obstructive pulmonary disease (COPD), pneumonia, asthma, bronchiectasis and interstitial lung disease—face substantial challenges in clinical recovery, including ventilatory weaning, restoration of gas exchange and prevention of respiratory failure (1, 2). In this context, psychological distress (particularly anxiety) and sedative/psychotropic medication exposure are common yet under-recognized factors that may negatively influence recovery trajectories (3).

First, psychological distress is increasingly acknowledged as a key co-morbidity in respiratory disease (4). Anxiety and dyspnoea frequently co-occur in COPD and other chronic lung conditions, leading not only to worse quality of life but also to impaired physiological functioning and prolonged hospitalization (5). For example, controlled breathing and relaxation techniques (as a non-pharmacological intervention) have been shown to reduce anxiety and improve dyspnoea, inspiratory pressures and mobility in hospitalized COPD patients (6). In addition, broader relaxation training such as mindfulness or guided imagery has been associated with lower respiratory rate, improved perception of dyspnoea and reduced anxiety in respiratory patients (7). These findings provide a rationale for considering psychological relaxation as a modifiable adjunct in respiratory care.

Second, sedative and psychotropic medication use—particularly early in hospitalization—may impose additional risk on respiratory recovery (8). Agents such as benzodiazepines, and combinations with opioids or other central nervous system depressants, have been linked to poorer respiratory outcomes (e.g., increased risk of respiratory failure, hypoventilation, higher mortality) in COPD and other populations (9). Mechanistically, benzodiazepines may depress respiratory drive (reducing ventilatory response to CO_2_), impair airway patency and blunt arousal to hypoxia or hypercapnia (10). In patients already compromised by gas-exchange impairment or ventilatory support dependence, this can translate into delayed recovery or higher likelihood of failure (11).

Despite this dual relevance of psychological state and pharmacologic sedation to respiratory outcomes, few studies have simultaneously examined the interplay of psychological relaxation interventions and sedative medication use in hospitalized patients with respiratory diseases (12). Specifically: (1) Does the implementation of relaxation training during hospitalization associate with higher odds of successful respiratory recovery at discharge? (2) Does early exposure to sedative or psychotropic medications (for example, benzodiazepines within the first 72 h) associate with worse recovery outcomes? And (3) how might both psychological and physiological parameters be jointly integrated into a predictive model for respiratory recovery?

Accordingly, in this retrospective observational cohort study of 389 adults hospitalized with primary respiratory diagnoses at a tertiary hospital in Sichuan Province between January 2020 and January 2025, we aimed to: (i) assess the independent association of documented relaxation training during hospitalization with respiratory recovery at discharge; (ii) examine the association of early sedative/psychotropic medication use (within 72 h of admission) with respiratory recovery; and (iii) construct and validate a multivariable logistic regression prediction model incorporating demographic (e.g., age), psychological (anxiety scores), physiological (PaCO_2_, PaO_2_/FiO_2_, CRP) and treatment-related (relaxation training, sedative use) factors. We hypothesized that participation in relaxation training would be positively associated with respiratory recovery, while early sedative medication exposure would be negatively associated, and that a combined model would offer clinically useful discrimination and calibration for guiding in-hospital decision-making.

## Materials and methods

### Study design and setting

This study employed a retrospective observational design based on routinely collected clinical data. All data were extracted from the electronic medical record system of Hejiang County People’s Hospital (Sichuan Province, China). The study population consisted of patients who had been admitted with a confirmed diagnosis of respiratory disease between January 1, 2020, and January 31, 2025. No interventions or follow-up procedures were performed by the investigators, and all exposures and outcomes had occurred prior to data collection.

The primary objective was to examine the association between relaxation training, psychotropic or sedative medication use, and respiratory recovery outcomes in hospitalized patients with respiratory diseases. Data collection and statistical analyses were performed retrospectively between March and June 2025 using de-identified patient information. The study protocol was reviewed and approved by the Ethics Committee of Hejiang County People’s Hospital, and the requirement for informed consent was waived owing to the retrospective design. All procedures were conducted in accordance with the Declaration of Helsinki.

### Participants

All eligible participants were identified retrospectively from the electronic medical record database of Hejiang County People’s Hospital. Inclusion criteria were: (1) age ≥ 18 years; and (2) hospitalization with a primary diagnosis of respiratory disease, including chronic obstructive pulmonary disease (COPD), pneumonia, asthma, bronchiectasis, or interstitial lung disease, as confirmed by attending physicians according to established clinical and imaging criteria.

Patients were excluded if they had (1) incomplete clinical or psychological assessment data; (2) documented severe neurological or psychiatric comorbidities that could confound anxiety evaluation; (3) terminal illness or do-not-resuscitate status at admission; or (4) inter-hospital transfer within 48 h of admission, resulting in incomplete hospitalization records.

Based on nursing and psychological documentation, patients were classified into two groups according to their recorded exposure to relaxation training during hospitalization. The “relaxation training” group comprised patients whose charts documented participation in standardized relaxation sessions delivered as part of routine nursing or psychological care, whereas the “non-relaxation” group had no such documentation. The relaxation techniques delivered in routine care primarily comprised breathing regulation, progressive muscle relaxation, and guided imagery. In our hospital, relaxation training was provided as brief, non-pharmacological bedside guidance integrated into routine nursing or psychosomatic care, rather than as a protocol-driven intervention. Documentation of relaxation training in the electronic medical record was not uniform; when recorded, notes typically indicated that a relaxation intervention had been delivered, with limited detail regarding the specific technique, frequency, or duration. Relaxation training was implemented by ward nurses and/or psychosomatic medicine staff who had received routine departmental instruction in relaxation guidance, and no formalized or time-mandated protocol was applied across the study period. All exposures occurred as part of standard hospital practice rather than study-driven interventions. Because this was a retrospective study, the frequency and duration of relaxation training were not protocol-mandated; therefore, we operationalized exposure as any documented participation during hospitalization (yes/no) based on nursing/psychological notes. No randomization, allocation, or matching procedures were involved.

### Variables

All study variables were retrospectively obtained from the electronic medical records of Hejiang County People’s Hospital. The primary outcome was respiratory recovery at discharge. To reduce subjectivity, it was defined a priori using a standardized algorithm based on routinely documented records, requiring all of the following: (1) successful discontinuation of invasive or non-invasive ventilatory support (if applicable); (2) stable oxygen saturation ≥ 95% on room air maintained for ≥ 4 prior to discharge; (3) respiratory rate 12–24 breaths/min at rest within the last 24 h before discharge; and (4) no evidence of ongoing respiratory failure, operationalized as pH ≥ 7.35 and no worsening hypercapnia (PaCO_2_ not increasing compared with the most recent prior measurement) and/or PaO_2_ ≥ 60 mmHg on room air when an arterial blood gas test was available within 24 h before discharge. When discharge-time arterial blood gas data were unavailable, criteria (1)–(3) and clinician-documented absence of respiratory failure were used. The main exposure variables included documented participation in relaxation training during hospitalization and sedative or psychotropic medication exposure. Sedative and psychotropic medication use was identified retrospectively from medication orders, nursing medication administration records, and pharmacy dispensing records. Due to incomplete and non-uniform documentation of dose, duration, administration route, and clinical indication in routine care records, sedative exposure was operationalized as a binary variable (yes/no) for each medication class. Early benzodiazepine exposure was defined a priori as any benzodiazepine administration within the first 72 h after admission as recorded in the medication administration record (yes/no). Clinical indications for sedative/psychotropic use were abstracted from physician orders and progress notes when explicitly documented (e.g., anxiety, insomnia, agitation, or intolerance of respiratory support), but indication fields were not consistently available for all administrations; therefore, indication was not modeled as an analytic covariate and is addressed as a limitation. Anxiety levels were assessed using the Hospital Anxiety and Depression Scale–Anxiety subscale (HADS-A), which was routinely administered during hospitalization as part of standard psychosomatic screening by ward nurses and/or psychosomatic medicine staff. Physiologic and laboratory variables collected at admission comprised age, sex, body mass index (BMI), current smoking status, arterial pH, arterial partial pressure of carbon dioxide (PaCO_2_), the ratio of arterial oxygen partial pressure to inspired oxygen fraction (PaO_2_/FiO_2_), C-reactive protein (CRP), and neutrophil-to-lymphocyte ratio (NLR). This time window was selected because it represents a clinically vulnerable period for respiratory compromise and ventilatory dependence. Information on benzodiazepine exposure was derived from medication administration records and pharmacy dispensing logs; however, detailed data on cumulative dose, duration of use, administration route, and clinical indication were not consistently available across patients and were therefore not included in quantitative analyses. To account for baseline clinical status, additional variables such as disease severity and ventilatory modality at admission (non-invasive or invasive ventilation) were also extracted. All variables were defined a priori, and data sources and measurement methods were consistent across participants to ensure comparability and internal validity.

### Data sources and measurements

All variables were retrieved from the hospital’s electronic medical record system and verified independently by two researchers. Physiological and laboratory measurements corresponded to the first evaluation within 24 h of admission. Outcome status at discharge was abstracted independently by two investigators using the prespecified criteria above, based on discharge summaries, nursing vital-sign charts, ventilatory support records, and the last available arterial blood gas results. Discrepancies were resolved by consensus with a senior respiratory clinician to ensure consistent adjudication. Inter-rater agreement for key variables, including exposure classification (relaxation training and benzodiazepine use) and outcome ascertainment, was assessed during data extraction, and substantial agreement was achieved (Cohen’s κ > 0.80), supporting the reliability of the extracted data. Medication records were cross-checked using the pharmacy information system to ensure accuracy. Because medication administration details were variably recorded in routine practice, exposure classification was based on the presence or absence of documented administration within prespecified time windows, rather than on dose- or duration-based metrics. Anxiety scores were derived from standardized HADS forms recorded during hospitalization; when multiple assessments were available, the earliest recorded HADS-A score was used for analysis. Relaxation training documentation was abstracted from structured nursing records and psychosomatic care notes, and exposure coding (yes/no) was performed using a prespecified abstraction guide to ensure consistent interpretation across reviewers. Data completeness was verified prior to analysis. Missing values were confined to a small proportion of routinely collected continuous laboratory variables (including PaCO_2_, PaO_2_/FiO_2_ ratio, CRP, and NLR), with no single variable exceeding a missing rate of 5%. Given the low proportion of missingness and the absence of evidence for systematic patterns, mean imputation was applied to continuous variables to minimize case loss and preserve statistical power in multivariable analyses.

### Bias control

To minimize selection bias, we identified consecutively admitted eligible patients within a prespecified observation window (January 1, 2020 to January 31, 2025) using standardized EMR queries and uniform inclusion/exclusion criteria. We recognize that EMR-based screening may still incompletely capture milder cases if key documentation elements are missing; this potential residual selection bias is acknowledged as a limitation. Because the primary objective was to examine associations and develop a clinically usable prediction model rather than estimate a causal treatment effect, and because baseline characteristics between groups were broadly comparable (Table 1), we did not apply propensity-score matching or weighting; instead, we addressed measured imbalances through multivariable adjustment with clinically prespecified covariates. Information bias was reduced through independent double abstraction with prespecified coding rules, cross-validation between EMR and pharmacy modules, and consensus adjudication procedures as described in the Data sources and measurements section.

**TABLE 1:** Baseline characteristics of 389 hospitalized patients with respiratory diseases, grouped by relaxation training.

Variables	Total (*n* = 389)	Relaxation training (*n* = 158)	No relaxation training (*n* = 231)	*P*-value
Age, years	63.3 ± 12.2	62.1 ± 11.8	64.2 ± 12.5	0.13
Male, *n* (%)	226 (58.1)	90 (57.0)	136 (58.9)	0.73
Body mass index, kg/m^2^	24.0 ± 3.8	23.9 ± 3.7	24.1 ± 3.9	0.61
Current smoker, n (%)	153 (39.3)	65 (41.1)	88 (38.1)	0.57
Primary diagnosis, n (%)	0.65 †			
Chronic obstructive pulmonary disease (COPD)	195 (50.1)	74 (46.8)	121 (52.4)	
Pneumonia	118 (30.3)	48 (30.4)	70 (30.3)	
Asthma	34 (8.7)	16 (10.1)	18 (7.8)	
Bronchiectasis	19 (4.9)	9 (5.7)	10 (4.3)	
Interstitial lung disease	23 (5.9)	11 (7.0)	12 (5.2)	
HADS-A score	10.8 ± 3.8	10.4 ± 3.6	11.2 ± 3.9	0.05
Arterial pH	7.36 ± 0.07	7.37 ± 0.06	7.36 ± 0.07	0.19
PaCO_2_, mmHg	50.5 ± 9.9	49.8 ± 9.4	51.1 ± 10.2	0.26
PaO_2_/FiO_2_ ratio	260 ± 75	268 ± 72	255 ± 76	0.17
C-reactive protein, mg/L	40 ± 30	38 ± 29	42 ± 31	0.28
Neutrophil-to-lymphocyte ratio	6.1 ± 3.6	5.9 ± 3.4	6.3 ± 3.8	0.32
Benzodiazepine use within 72 h, n (%)	120 (30.8)	42 (26.6)	78 (33.8)	0.13
Dexmedetomidine use, n (%)	39 (10.0)	14 (8.9)	25 (10.8)	0.59
Opioid use during hospitalization, n (%)	53 (13.6)	19 (12.0)	34 (14.7)	0.47
Systemic corticosteroid use, n (%)	241 (62.0)	94 (59.5)	147 (63.6)	0.45
Non-invasive ventilation at admission, n (%)	75 (19.3)	27 (17.1)	48 (20.8)	0.38
Invasive mechanical ventilation at admission, n (%)	25 (6.4)	9 (5.7)	16 (6.9)	0.67

HADS-A, Hospital Anxiety and Depression Scale-Anxiety subscale; PaCO_2_, arterial partial pressure of carbon dioxide; PaO_2_/FiO_2_, ratio of arterial oxygen partial pressure to inspired oxygen fraction; CRP, C-reactive protein; NLR, neutrophil-to-lymphocyte ratio; COPD, chronic obstructive pulmonary disease. Continuous variables are presented as mean ± standard deviation, and categorical variables as number (percentage).

### Study size

A total of 389 patients met all inclusion criteria after screening 428 hospitalized cases. The sample size was determined by the number of eligible patients available within the study period and was sufficient to provide at least ten outcome events per variable in multivariable logistic regression, ensuring model stability.

### Quantitative variables and data handling

Continuous variables were summarized as mean ± standard deviation (SD), and categorical variables as number (percentage). For regression analyses, age, HADS-A score, PaCO_2_, PaO_2_/FiO_2_ ratio, and CRP were retained as continuous predictors to preserve information. The PaO_2_/FiO_2_ ratio was scaled per 10-unit increase for interpretability. No arbitrary categorization was used unless clinically justified.

### Statistical analysis

All statistical analyses were conducted using SPSS version 26.0 (IBM Corp., Armonk, NY, United States) and R version 4.3.2. Continuous variables were expressed as mean ± standard deviation. Prior to between-group comparisons, the normality of continuous variables was assessed; independent-sample *t*-tests were applied when distributional assumptions were met, whereas categorical variables were summarized as frequencies and percentages and compared using the χ^2^-test. To identify factors associated with respiratory recovery, univariate logistic regression analyses were first performed for candidate predictors. Variables were selected for the multivariable model primarily based on prespecified clinical relevance and prior evidence, with additional consideration of variables showing an association at *P* < 0.10 in univariate analyses; all selected variables were entered simultaneously using the enter method. Adjusted odds ratios (ORs) and corresponding 95% confidence intervals (CIs) were calculated to estimate the strength and direction of associations. The overall model fit was assessed by the likelihood-ratio test, and model discrimination was evaluated by calculating the concordance index (C-index), which is equivalent to the area under the receiver operating characteristic curve (AUC). Model calibration was examined using the Hosmer–Lemeshow goodness-of-fit test and visualized with a calibration curve, while clinical interpretability was enhanced through the construction of a nomogram derived from the final regression coefficients. In addition, receiver operating characteristic analysis and decision curve analysis were applied to assess the discriminative ability and clinical utility of the predictive model. All statistical tests were two-tailed, and a *P*< 0.05 was considered statistically significant.

## Results

### Baseline characteristics

A total of 389 hospitalized patients with respiratory diseases were included, comprising 158 patients who received relaxation training and 231 who did not. The mean age of the study population was 63.3 ± 12.2 years, with no significant age difference between the two groups (62.1 ± 11.8 years vs. 64.2 ± 12.5 years, *P* = 0.13). Males accounted for 58.1% of all participants, and the gender distribution was comparable between the relaxation and non-relaxation groups (57.0% vs. 58.9%, *P* = 0.73). The mean body mass index was 24.0 ± 3.8 kg/m^2^, and the proportion of current smokers was 39.3%, with no significant intergroup differences. Regarding the primary diagnoses, chronic obstructive pulmonary disease (COPD) was the most common condition (50.1%), followed by pneumonia (30.3%), asthma (8.7%), bronchiectasis (4.9%), and interstitial lung disease (5.9%). The distribution of underlying respiratory diseases did not differ significantly between groups (*P* = 0.65). Patients who underwent relaxation training exhibited slightly lower anxiety scores than those without such training (HADS-A: 10.4 ± 3.6 vs. 11.2 ± 3.9, *P* = 0.05). No significant differences were observed in baseline arterial pH (7.37 ± 0.06 vs. 7.36 ± 0.07, *P* = 0.19), PaCO_2_ (49.8 ± 9.4 mmHg vs. 51.1 ± 10.2 mmHg, *P* = 0.26), or PaO_2_/FiO_2_ ratio (268 ± 72 vs. 255 ± 76, *P* = 0.17). Inflammatory markers were also similar between groups, with mean CRP levels of 38 ± 29 mg/L in the relaxation group and 42 ± 31 mg/L in the non-relaxation group (*P* = 0.28), and mean NLR values of 5.9 ± 3.4 vs. 6.3 ± 3.8 (*P* = 0.32). In terms of medication use, benzodiazepine administration within 72 h of admission was slightly less frequent in the relaxation group than in the non-relaxation group (26.6% vs. 33.8%, *P* = 0.13), while the use of dexmedetomidine (*P* = 0.59), opioids (*P* = 0.47), and systemic corticosteroids (*P* = 0.45) was comparable between groups. Similarly, the proportions of patients requiring non-invasive ventilation (17.1% vs. 20.8%, *P* = 0.38) or invasive mechanical ventilation at admission (5.7% vs. 6.9%, *P* = 0.67) showed no statistically significant difference. Overall, the two groups were well balanced in demographic, clinical, and laboratory characteristics, except for a trend toward lower anxiety levels and reduced benzodiazepine use among patients who received relaxation training. The results of the univariate analyses are presented in Table 2, and the multivariable logistic regression results are shown in Table 3.

**TABLE 2:** Univariate analysis of factors associated with respiratory recovery among 389 hospitalized patients with respiratory diseases.

Variables	Recovery achieved (*n* = 245)	Recovery not achieved (*n* = 144)	Odds ratio (95% CI)	*P*-value
Age, years	61.7 ± 11.9	66.0 ± 12.5	0.97 (0.95–0.99)	0.004
Male, n (%)	146 (59.6)	80 (55.6)	1.17 (0.77–1.78)	0.46
Body mass index, kg/m^2^	24.1 ± 3.7	23.8 ± 3.9	1.02 (0.96–1.09)	0.53
Current smoker, n (%)	87 (35.5)	66 (45.8)	0.65 (0.43–0.99)	0.045
COPD, n (%)	108 (44.1)	87 (60.4)	0.52 (0.34–0.80)	0.003
Pneumonia, n (%)	78 (31.8)	40 (27.8)	1.21 (0.77–1.91)	0.40
Asthma, n (%)	26 (10.6)	8 (5.6)	1.99 (0.86–4.59)	0.11
Bronchiectasis, n (%)	12 (4.9)	7 (4.9)	1.00 (0.38–2.63)	0.99
Interstitial lung disease, n (%)	9 (3.7)	14 (9.7)	0.36 (0.15–0.83)	0.016
HADS-A score	9.9 ± 3.4	12.0 ± 3.9	0.86 (0.80–0.92)	< 0.001
Arterial pH	7.37 ± 0.06	7.34 ± 0.07	3.62 (1.77–7.40) *	< 0.001
PaCO_2_, mmHg	48.7 ± 9.1	54.0 ± 10.1	0.92 (0.89–0.95)	< 0.001
PaO_2_/FiO_2_ ratio	274 ± 73	235 ± 78	1.01 (1.00–1.01)	< 0.001
CRP, mg/L	36 ± 27	49 ± 34	0.98 (0.97–0.99)	0.002
NLR	5.3 ± 3.1	7.2 ± 4.0	0.91 (0.86–0.96)	< 0.001
Relaxation training, n (%)	120 (49.0)	38 (26.4)	2.61 (1.64–4.15)	< 0.001
Benzodiazepine use within 72 h, n (%)	57 (23.3)	63 (43.8)	0.39 (0.25–0.61)	< 0.001
Dexmedetomidine use, n (%)	19 (7.8)	20 (13.9)	0.52 (0.27–0.98)	0.042
Opioid use during hospitalization, n (%)	25 (10.2)	28 (19.4)	0.47 (0.26–0.85)	0.012
Systemic corticosteroid use, n (%)	141 (57.6)	100 (69.4)	0.60 (0.39–0.93)	0.021
Non-invasive ventilation at admission, n (%)	32 (13.1)	43 (29.9)	0.35 (0.21–0.59)	< 0.001
Invasive mechanical ventilation at admission, n (%)	7 (2.9)	18 (12.5)	0.21 (0.09–0.50)	< 0.001

HADS-A, Hospital Anxiety and Depression Scale–Anxiety subscale; PaCO_2_, arterial partial pressure of carbon dioxide; PaO_2_/FiO_2_, ratio of arterial oxygen partial pressure to inspired oxygen fraction; CRP, C-reactive protein; NLR, neutrophil-to-lymphocyte ratio; COPD, chronic obstructive pulmonary disease. Values are presented as mean ± standard deviation for continuous variables and number (percentage) for categorical variables. Odds ratios (ORs) and 95% confidence intervals were calculated by univariate logistic regression, with *P* < 0.05 considered statistically significant.

**TABLE 3:** Multivariable logistic regression analysis of independent predictors of respiratory recovery.

Variables	β coefficient (SE)	Odds ratio (95% CI)	*P*-value
Age, per year increase	–0.036 (0.012)	0.96 (0.94–0.98)	0.002
HADS-A score	–0.145 (0.037)	0.87 (0.81–0.93)	<0.001
PaCO_2_, mmHg	–0.058 (0.015)	0.94 (0.91–0.97)	<0.001
PaO_2_/FiO_2_ ratio, per 10-unit increase	0.072 (0.021)	1.07 (1.03–1.12)	0.001
CRP, mg/L	–0.019 (0.007)	0.98 (0.97–0.99)	0.007
Relaxation training (yes vs. no)	0.868 (0.255)	2.38 (1.45–3.90)	<0.001
Benzodiazepine use within 72 h (yes vs. no)	–0.982 (0.272)	0.37 (0.22–0.63)	<0.001
Non-invasive ventilation at admission (yes vs. no)	–0.971 (0.312)	0.38 (0.21–0.69)	0.001

HADS-A, Hospital Anxiety and Depression Scale–Anxiety subscale; PaCO_2_, arterial partial pressure of carbon dioxide; PaO_2_/FiO_2_, ratio of arterial oxygen partial pressure to inspired oxygen fraction; CRP, C-reactive protein. Variables with *P* < 0.10 in univariate analysis or of clinical importance were entered into a multivariable logistic regression model using the enter method. Adjusted odds ratios (ORs) with 95% confidence intervals are presented.

### Univariate analysis of factors associated with respiratory recovery

In the univariate analysis, several demographic, clinical, psychological, and pharmacological factors were found to be associated with respiratory recovery among 389 hospitalized patients with respiratory diseases. Patients who achieved recovery (n = 245) were significantly younger than those who did not (61.7 ± 11.9 vs. 66.0 ± 12.5 years, *P* = 0.004), and current smoking was less common in the recovery group (35.5% vs. 45.8%, *P* = 0.045). The distribution of primary respiratory diagnoses also differed: recovery was more frequent among patients with COPD (44.1% vs. 60.4%, *P* = 0.003) and less frequent among those with interstitial lung disease (3.7% vs. 9.7%, *P* = 0.016).

Psychological status showed a strong association with clinical outcomes. Patients who recovered had significantly lower anxiety scores than those who did not (HADS-A: 9.9 ± 3.4 vs. 12.0 ± 3.9, *P* < 0.001). Regarding physiological parameters, favorable acid–base balance and gas exchange were observed in the recovery group, characterized by higher arterial pH (7.37 ± 0.06 vs. 7.34 ± 0.07, *P* < 0.001), lower PaCO_2_ (48.7 ± 9.1 vs. 54.0 ± 10.1 mmHg, *P* < 0.001), and higher PaO_2_/FiO_2_ ratios (274 ± 73 vs. 235 ± 78, *P* < 0.001). Inflammatory activity was milder among recovered patients, as reflected by lower CRP (36 ± 27 vs. 49 ± 34 mg/L, *P* = 0.002) and NLR values (5.3 ± 3.1 vs. 7.2 ± 4.0, *P* < 0.001). Relaxation training was strongly associated with better recovery outcomes (49.0% vs. 26.4%, *P* < 0.001), whereas the use of benzodiazepines within 72 h of admission was linked to poorer recovery (23.3% vs. 43.8%, *P* < 0.001). Similarly, patients who recovered were less likely to receive dexmedetomidine (*P* = 0.042), opioids (*P* = 0.012), or systemic corticosteroids (*P* = 0.021). The need for ventilatory support at admission also indicated worse prognosis: non-invasive ventilation (13.1% vs. 29.9%, *P* < 0.001) and invasive mechanical ventilation (2.9% vs. 12.5%, *P* < 0.001) were both significantly more frequent in patients who failed to recover. Overall, younger age, lower anxiety scores, better gas-exchange parameters, lower inflammatory burden, absence of early sedative use, and exposure to relaxation training were all associated with an increased likelihood of respiratory recovery in univariate analysis.

### Multivariable analysis of independent predictors of respiratory recovery

In the multivariable logistic regression analysis, several independent predictors of respiratory recovery were identified after adjusting for potential confounders. Younger age remained a significant protective factor, with each additional year associated with a 4% decrease in the odds of recovery (OR = 0.96, 95% CI 0.94–0.98, *P* = 0.002). Psychological status also played an important role: higher anxiety levels, as indicated by HADS-A scores, were independently associated with poorer recovery outcomes (OR = 0.87, 95% CI 0.81–0.93, *P* < 0.001). Among physiological parameters, higher PaCO_2_ levels were strongly linked to reduced odds of recovery (OR = 0.94, 95% CI 0.91–0.97, *P* < 0.001), whereas a higher PaO_2_/FiO_2_ ratio predicted a greater likelihood of improvement (OR = 1.07 per 10-unit increase, 95% CI 1.03–1.12, *P* = 0.001). Inflammatory status also contributed significantly: elevated CRP concentrations were inversely associated with recovery (OR = 0.98, 95% CI 0.97–0.99, *P* = 0.007). Importantly, participation in relaxation training emerged as a strong positive predictor of respiratory recovery (OR = 2.38, 95% CI 1.45–3.90, *P* < 0.001), suggesting that psychological relaxation interventions may facilitate clinical improvement. In contrast, early benzodiazepine exposure within 72 h of admission was independently associated with a 63% reduction in the likelihood of recovery (OR = 0.37, 95% CI 0.22–0.63, *P* < 0.001). Additionally, the need for non-invasive ventilation on admission significantly decreased the probability of recovery (OR = 0.38, 95% CI 0.21–0.69, *P* = 0.001). Collectively, these findings indicate that a combination of demographic, psychological, physiological, and treatment-related factors independently influence respiratory recovery. Specifically, lower anxiety levels, better gas-exchange parameters, reduced systemic inflammation, and engagement in relaxation training were all associated with a higher probability of successful recovery, while early sedative use and ventilatory dependence predicted unfavorable outcomes.

### Model performance

**FIGURE 1:**
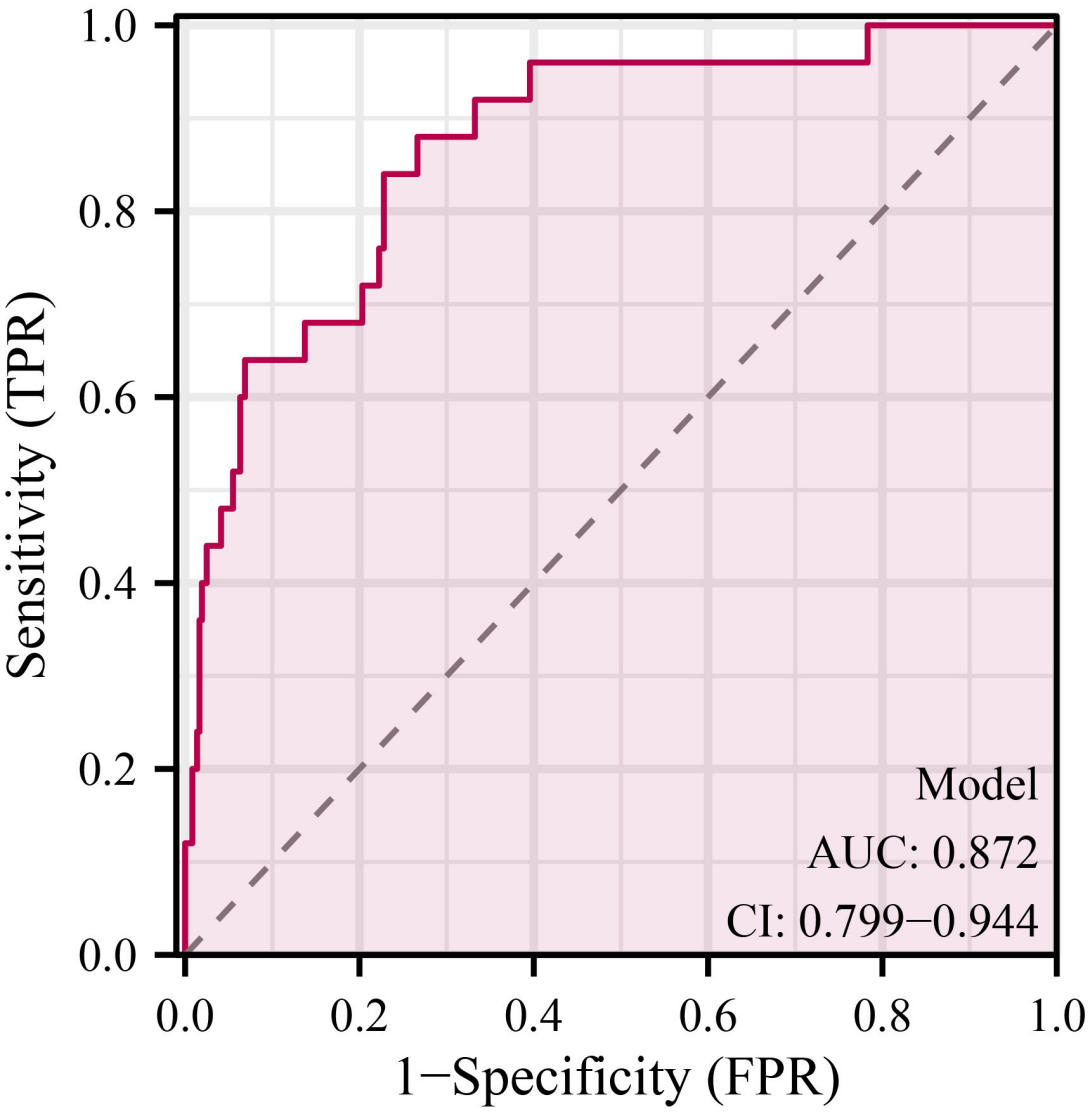
Receiver operating characteristic (ROC) curve of the prediction model. The ROC curve demonstrates the discriminative performance of the final logistic regression model for predicting respiratory recovery in patients with respiratory diseases. The area under the curve (AUC) was 0.872 (95% CI: 0.799–0.944), indicating good discrimination. The diagonal gray dashed line represents a reference line with no discrimination (AUC = 0.5).

**FIGURE 2:**
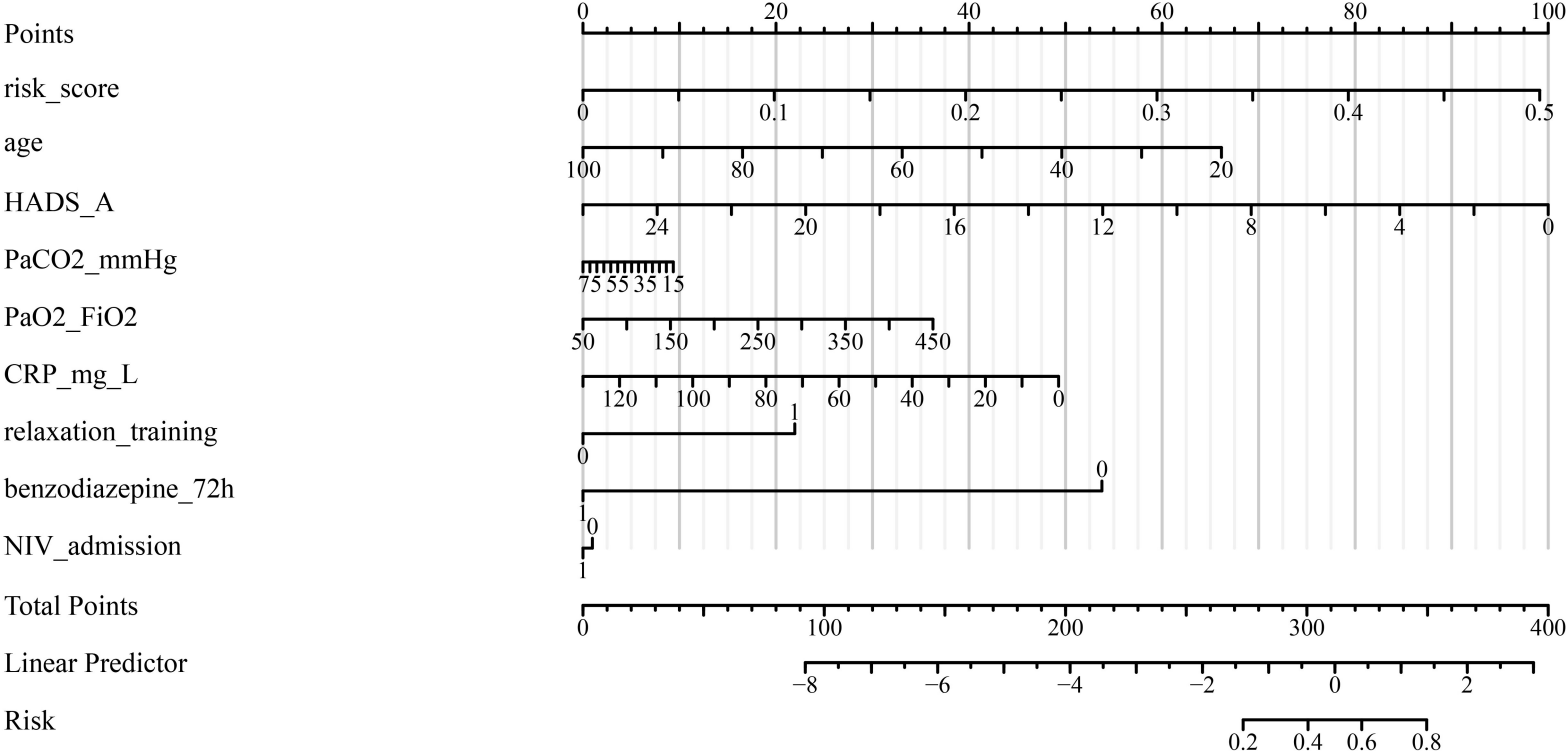
Calibration plot of the prediction model. The calibration plot compares the predicted and observed probabilities of respiratory recovery. The blue line represents the apparent performance of the model, the red line represents the bias-corrected performance after 1000 bootstrap resamples, and the gray dashed line indicates perfect calibration. The Hosmer–Lemeshow goodness-of-fit test showed no significant deviation between predicted and observed probabilities (χ^2^ = 6.585, *P* = 0.582), suggesting satisfactory calibration.

**FIGURE 3:**
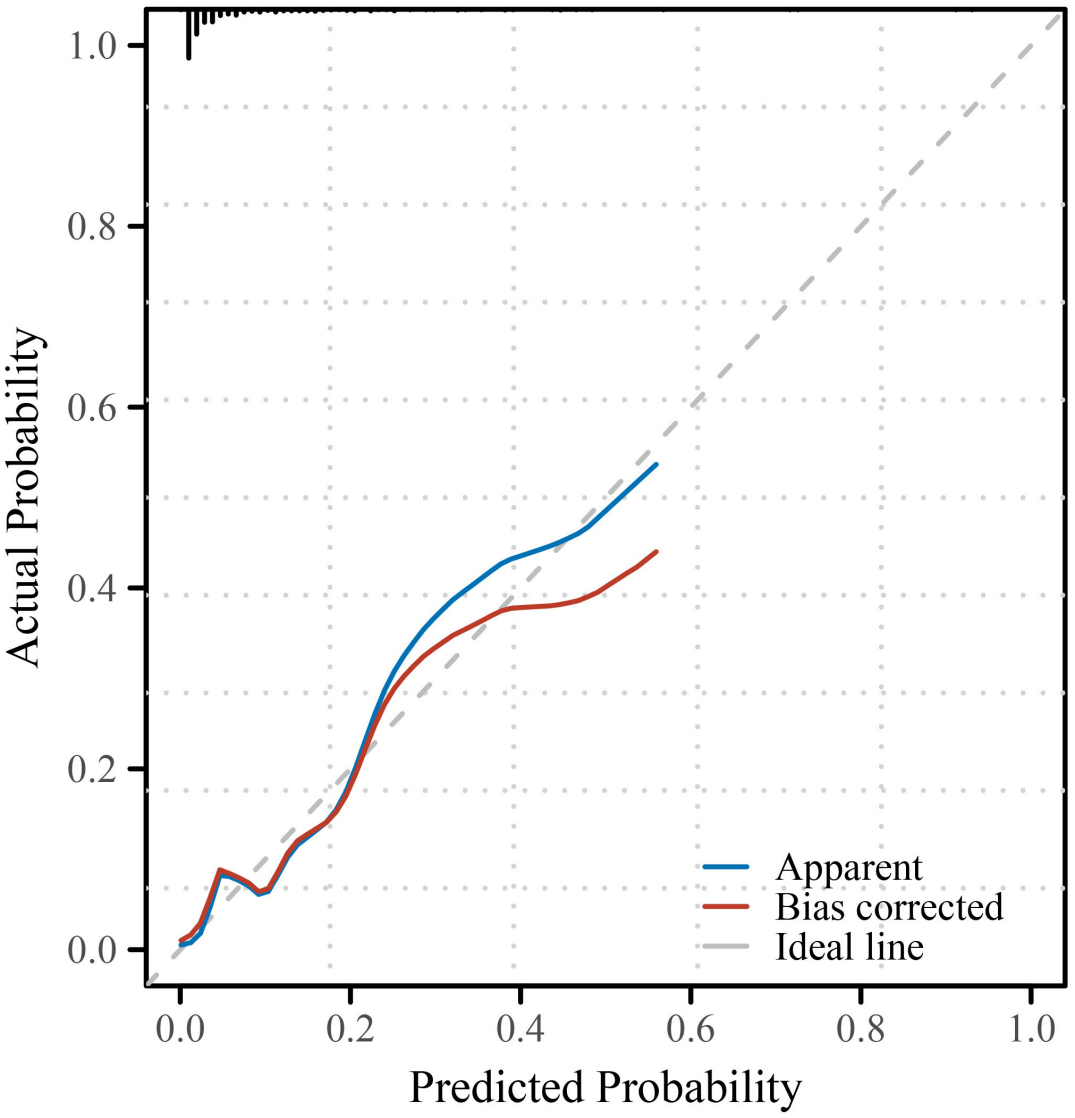
Nomogram for predicting respiratory recovery. The nomogram was constructed based on independent predictors identified in multivariable logistic regression analysis, including age, HADS-A score, PaCO_2_, PaO_2_/FiO_2_ ratio, CRP, relaxation training, benzodiazepine use, and non-invasive ventilation. Each variable corresponds to a specific point score, and the total points correspond to an estimated probability of recovery.

**FIGURE 4:**
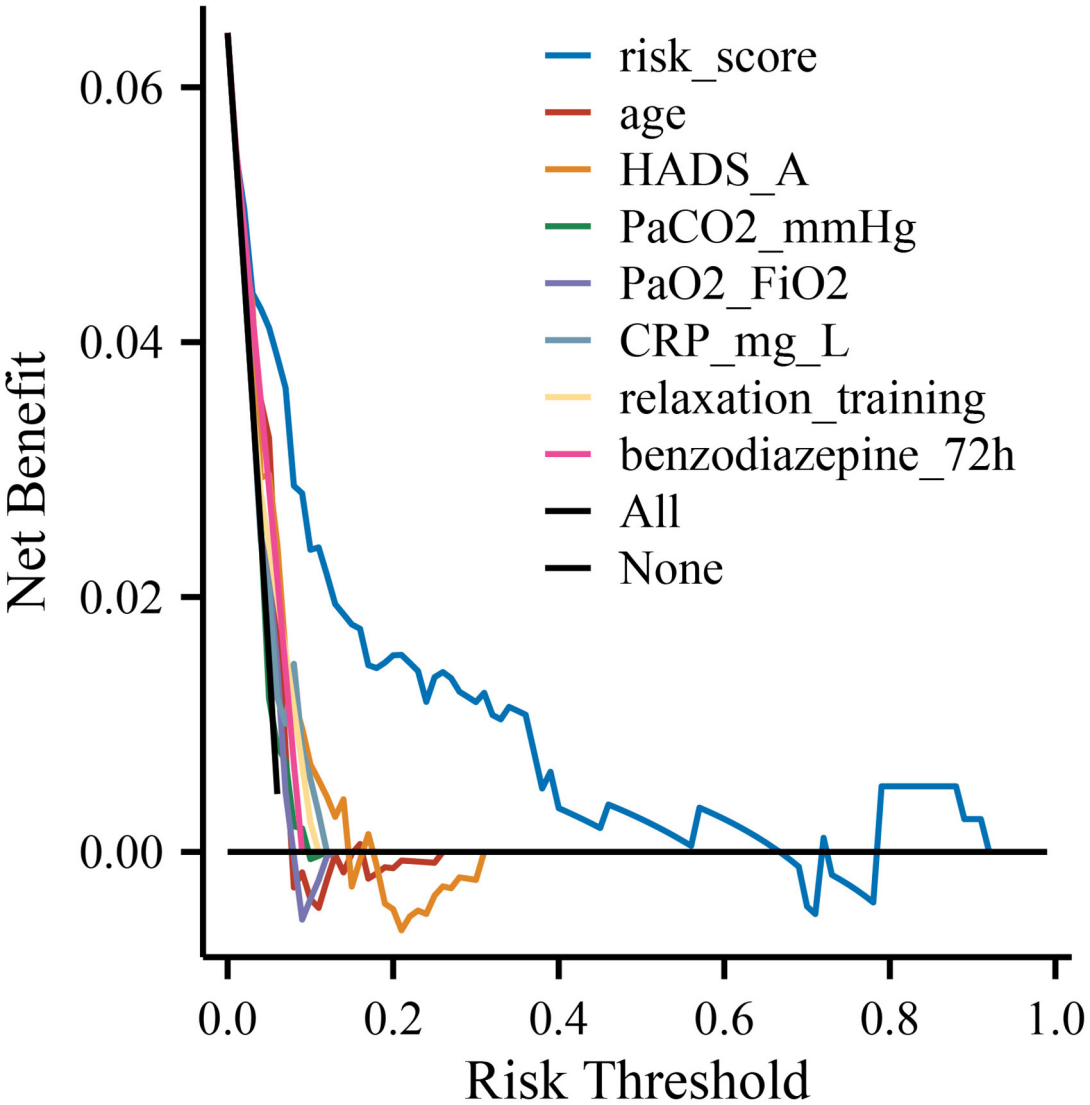
Decision curve analysis (DCA) for clinical utility of the model. The DCA curve illustrates the net clinical benefit of the prediction model across a range of threshold probabilities. The blue line represents the model, the gray line represents the “treat all” strategy, and the black line represents the “treat none” strategy. The model provided higher net benefit across clinically relevant probability thresholds, indicating favorable clinical applicability.

As shown in Table 4, the overall model fit was statistically significant by the likelihood ratio test (χ^2^ = 53.783, *P* = 2.08 × 10^–8^), indicating that at least one predictor contributed independently to the outcome. The model demonstrated good discrimination, with a C-index (AUC) of 0.872 (95% CI, 0.799–0.944; *P* = 1.38 × 10^–24^), which is visually corroborated by the ROC curve where the empirical trajectory lies well above the reference line (AUC = 0.5) (Figure 1). Calibration was satisfactory: the Hosmer–Lemeshow goodness-of-fit test showed no evidence of lack of fit (χ^2^ = 6.585, *P* = 0.582), and the bias-corrected calibration curve closely tracked the ideal 45° line across the probability range (Figure 2). At the optimal cut-off (–2.9804), the model achieved a sensitivity of 0.880, specificity of 0.734, overall accuracy of 0.743, PPV of 0.185, NPV of 0.989, and a Youden index of 0.614 (Table 4), indicating strong ruling-out capacity with high negative predictive value. To enhance clinical interpretability, we constructed a nomogram mapping each independent predictor to a point scale with a corresponding predicted probability of recovery (Figure 3). Finally, decision curve analysis demonstrated that the model yields a consistently higher net benefit than the “treat-all” and “treat-none” strategies across clinically relevant threshold probabilities, supporting its potential clinical utility (Figure 4).

**TABLE 4:** Model performance and validation metrics.

Evaluation index	Statistical content	Statistic (95% CI)	*P-*value
Model test	Likelihood ratio test	χ^2^ = 53.783	2.08 × 10^–8^
Discrimination ability	C-index (AUC)	0.872 (0.799–0.944)	1.38 × 10^–24^
Calibration ability	Hosmer–Lemeshow goodness-of-fit test	χ^2^ = 6.585	0.582
Optimal cut-off value	–2.9804	–	–
Sensitivity	–	0.880	–
Specificity	–	0.734	–
Accuracy	–	0.743	–
Positive predictive value (PPV)	–	0.185	–
Negative predictive value (NPV)	–	0.989	–
Youden index	–	0.614	–

The model demonstrated strong overall significance by the likelihood ratio test (*P* < 0.001). Discrimination was good (C-index = 0.872, 95% CI 0.799–0.944), and the calibration was satisfactory according to the Hosmer–Lemeshow test (*P* = 0.582). At the optimal cut-off (–2.9804), the model achieved a sensitivity of 88.0% and specificity of 73.4%.

## Discussion

In this retrospective cohort of 389 hospitalized adults with respiratory diseases, we found that participation in relaxation training during hospitalization was independently associated with a higher probability of respiratory recovery at discharge, whereas early exposure to benzodiazepines within 72 h was associated with a substantially lower likelihood of recovery after multivariable adjustment for demographic, psychological, physiological and treatment-related factors. These findings highlight the dual and potentially opposing influences of non-pharmacological psychological care and pharmacologic sedation on short-term respiratory outcomes in real-world practice.

Our observations regarding relaxation training align with accumulating evidence that breathing-focused and relaxation-oriented strategies can alleviate dyspnoea and anxiety and improve functional status in people with chronic respiratory conditions, including COPD (13, 14). A recent synthesis of breathing techniques—encompassing breathing retraining, relaxation and related modalities—suggests benefits across dyspnoea, anxiety and health-related quality of life, supporting the clinical plausibility of our findings in an inpatient setting (15). In addition, emerging trials of mindfulness-based or guided-imagery interventions in COPD demonstrate reductions in immediate anxiety and perceived breathlessness, reinforcing the role of broader relaxation training as a feasible, modifiable adjunct to usual respiratory care pathways (16, 17). While most prior trials have emphasized outpatient or digitally delivered formats, our results extend the relevance of relaxation training to hospitalized patients, in whom anxiety, immobilization and acute respiratory impairment often converge to jeopardize recovery (7, 18).

Conversely, our finding that early benzodiazepine exposure predicted worse recovery is consistent with contemporary data indicating potential harm from sedative or sedative-analgesic regimens in acute respiratory care, especially when used early and/or deeply (19). In patients receiving non-invasive ventilation for acute respiratory failure, observational evidence links sedative/analgesic exposure to higher risks of intubation or death, underscoring that even “light” sedation can unfavorably shift the risk-benefit balance in vulnerable respiratory phenotypes (12). In COPD populations, cohort data associate benzodiazepines and, particularly, combinations with opioids with higher mortality, suggesting that central nervous system depressants can exacerbate ventilatory compromise or blunt compensatory responses during exacerbations or advanced disease (9). Mechanistically, benzodiazepines reduce respiratory drive and dampen ventilatory responses to hypoxia and hypercapnia; they may also impair airway patency and arousal, thereby potentiating hypoventilation in patients with gas-exchange impairment or sleep-disordered breathing, which coheres with the direction of effect we observed for recovery failure (20). Taken together, these external data provide biological and clinical plausibility for our multivariable associations between early sedative exposure and poorer recovery. The physiological covariates retained by our model—lower PaCO_2_, higher PaO_2_/FiO_2_ and lower CRP—map onto canonical pathophysiology linking better gas exchange and lower systemic inflammation to favorable trajectories in acute-on-chronic respiratory illness (21). Notably, indices that capture ventilatory inefficiency or impaired gas exchange have been associated with extubation failure and prolonged intensive care unit (ICU) length of stay, providing external validity to our composite “recovery” outcome that required stability off ventilatory support and adequate oxygenation on room air (22). Importantly, anxiety burden (higher HADS-A) independently predicted poorer recovery in our cohort, resonating with the well-documented co-occurrence of anxiety and dyspnoea and their bidirectional amplification, which can increase perceived breathing effort, exacerbate dynamic hyperinflation, and impede mobilization and pulmonary rehabilitation during admission (23). In this light, relaxation training may operate through both psychological (reducing anxiety, catastrophizing and autonomic arousal) and physiological pathways (optimizing breathing patterns, improving inspiratory muscle coordination), thereby enhancing readiness for ventilatory weaning and discharge stability (24).

Our results have several practical implications. First, embedding structured relaxation content—such as progressive muscle relaxation, guided breathing and brief mindfulness exercises—within routine nursing care may represent a low-cost, scalable intervention to augment standard respiratory management during hospitalization, particularly for patients with elevated anxiety scores or early signs of dyspnoea-related distress. Second, our findings support judicious use of sedatives in the early hospitalization window for respiratory patients, with preferential emphasis on non-pharmacologic strategies for anxiety or agitation when clinically safe, and careful daily reassessment of indication, depth and duration when pharmacologic agents are unavoidable. Third, the predictive model showed high discrimination and sound calibration, and its nomogram may assist clinicians in individualized risk estimation and shared decision-making surrounding ventilatory weaning, early mobilization and discharge planning, although external validation is warranted before clinical deployment.

This study also offers methodological strengths. We prespecified clinically coherent variables, applied double-source verification of medication and physiological data, and evaluated model performance across discrimination, calibration and decision-analytic domains, which together strengthen internal validity and potential clinical interpretability. Furthermore, by concurrently analyzing psychological (HADS-A), physiological (PaCO_2_, PaO_2_/FiO_2_) and treatment-related (relaxation training, sedative exposure) factors, we begin to bridge silos between behavioral and pharmacologic domains that are often studied separately in respiratory care.

Several limitations merit consideration when interpreting these findings. Because of the retrospective observational design, the associations observed in this study should not be interpreted as causal effects, but rather as correlations that may partly reflect underlying disease severity, patient characteristics, and clinical decision-making processes. Although outcome ascertainment was strengthened using prespecified criteria derived from routine records, some degree of inter-clinician variability and documentation-related misclassification cannot be fully excluded in this retrospective setting. First, the retrospective single-center design inherently limits causal inference and raises the possibility of residual confounding. Although we applied multivariable adjustment using clinically relevant covariates, unmeasured or incompletely captured factors—such as baseline cognitive status, pre-existing sleep disorders, or staff-patient interaction intensity—could still have influenced both the likelihood of receiving relaxation training and the decision to administer sedatives. In particular, confounding by indication remains plausible: patients who were more cooperative, motivated, or communicative may have been more likely to engage in relaxation sessions, whereas those with agitation, delirium, or advanced disease severity were more likely to receive benzodiazepines. In addition, some clinically important confounders—such as prior psychological treatment history, family support, and finer-grained severity stratification within disease subtypes—were not consistently available in structured EMR fields and could not be incorporated, which may contribute to residual confounding. Second, exposure to “relaxation training” was identified from nursing or psychological documentation within the routine electronic medical record. No standardized protocol existed for frequency, duration, or content of the intervention. Consequently, substantial heterogeneity in the fidelity and timing of relaxation practices could attenuate the measured association, biasing effect estimates toward the null. Future prospective studies should therefore employ a clearly defined intervention manual with dose recording, adherence tracking, and training certification for deliverers. Third, our pharmacologic variable focused specifically on early benzodiazepine use within the first 72 h, as this period represents the most vulnerable phase for ventilatory dependence. However, other sedative classes—such as propofol, dexmedetomidine, and opioid co-administration—were not examined in depth. We also lacked quantitative data on cumulative dose, duration, and sedation depth, preventing dose-response analyses that could better delineate risk gradients or temporal relationships. In addition, administration routes and clinical indications for sedative initiation were not uniformly documented in a structured format, which limited our ability to further characterize medication exposure beyond binary classification. Incorporating pharmacy dispensing logs and continuous sedation scoring in future cohorts would improve mechanistic interpretation. Fourth, although the primary endpoint—respiratory recovery at discharge—integrated clinically meaningful components (successful ventilator weaning, stable oxygenation on room air, and absence of respiratory failure symptoms), post-discharge trajectories were not assessed. It remains unknown whether in-hospital improvements persisted or translated into reduced readmissions, mortality, or long-term psychological outcomes. Prior research has demonstrated that persistent gas-exchange impairment and ventilatory dependence can predict long-term morbidity and impaired quality of life, highlighting the importance of extended follow-up and linkage with outpatient data systems. Finally, the external generalizability of our findings may be limited by site-specific practice patterns—including sedation protocols, nurse-to-patient ratios, and availability of psychological support services—as well as by the diagnostic composition of the cohort. Multicenter, prospective validation across different hospital levels and geographic regions will therefore be essential to confirm robustness and reproducibility.

Future studies should prospectively test structured, nurse-delivered relaxation protocols initiated early during hospitalization, with predefined frequency, duration and competency checks, and evaluate patient-centered outcomes (dyspnoea, anxiety trajectories), physiological endpoints (respiratory rate variability, inspiratory pressures) and care outcomes (weaning time, length of stay, readmissions). In parallel, pragmatic trials or high-quality registries should refine causal estimates for sedative exposure by incorporating time-varying dosing, depth of sedation, and co-administration with opioids, ideally leveraging analytic frameworks (e.g., marginal structural models) that better handle confounding by indication, as encouraged by recent NIV literature. Finally, hybrid designs could examine the additive or interactive effects of relaxation training and sedative-sparing strategies on recovery, addressing the current evidence gap on how behavioral and pharmacologic approaches jointly shape inpatient respiratory trajectories.

In conclusion, among hospitalized patients with respiratory diseases, relaxation training was independently associated with improved respiratory recovery, whereas early benzodiazepine exposure predicted poorer outcomes after adjustment for psychological and physiological covariates. These data, viewed alongside contemporary evidence on breathing-focused and mindfulness-based interventions and the risks of sedative exposure in respiratory care, support integrating structured relaxation practices and sedative stewardship into multidisciplinary inpatient pathways to optimize respiratory recovery.

## Data Availability

The original contributions presented in the study are included in the article/supplementary material, further inquiries can be directed to the corresponding author.
